# Effects of TP53 Mutations and miRs on Immune Responses in the Tumor Microenvironment Important in Pancreatic Cancer Progression

**DOI:** 10.3390/cells11142155

**Published:** 2022-07-09

**Authors:** James A. McCubrey, Li V. Yang, Stephen L. Abrams, Linda S. Steelman, Matilde Y. Follo, Lucio Cocco, Stefano Ratti, Alberto M. Martelli, Giuseppa Augello, Melchiorre Cervello

**Affiliations:** 1 Department of Microbiology and Immunology, Brody School of Medicine, East Carolina University, Greenville, NC 27858, USA; abramss@ecu.edu (S.L.A.); lssteelman@gmail.com (L.S.S.); 2 Department of Internal Medicine, Brody School of Medicine, East Carolina University, Greenville, NC 27858, USA; yangl@ecu.edu; 3 Dipartimento di Scienze Biomediche e Neuromotorie, Università di Bologna, 40139 Bologna, Italy; matilde.follo@unibo.it (M.Y.F.); lucio.cocco@unibo.it (L.C.); stefano.ratti@unibo.it (S.R.); alberto.martelli@unibo.it (A.M.M.); 4 Institute for Biomedical Research and Innovation, National Research Council (CNR), 90146 Palermo, Italy; giuseppa.augello@irib.cnr.it (G.A.); melchiorre.cervello@irib.cnr.it (M.C.)

**Keywords:** TP53, KRas, PDAC, immunotherapy, miRs, ncRNAs, tumor microenvironment, tumor stroma, targeted therapy, PD-L1

## Abstract

Approximately 90% of pancreatic cancers are pancreatic ductal adenocarcinomas (PDAC). PDAC is the fourth leading cause of cancer death world-wide. Therapies for PDAC are largely ineffective due to the dense desmoplastic tumor microenvironment which prevents chemotherapeutic drugs and small molecule inhibitors from exerting effective anti-cancer effects. In this review, we will discuss the roles of TP53 and miRs on the PDAC tumor microenvironment and how loss of the normal functions of TP53 promote tumor progression. The *TP53* gene is mutated in approximately 50% of pancreatic cancers. Often, these TP53 mutations are point mutations which confer additional functions for the TP53 proteins. These are called gain of function (GOF) mutations (mut). Another class of TP53 mutations are deletions which result in loss of the TP53 protein; these are referred to TP53-null mutations. We have organized this review into various components/properties of the PDAC microenvironment and how they may be altered in the presence of mutant TP53 and loss of certain miR expression.

## 1. Introduction-Overview of Genes Frequently Mutated in PDAC

There are multiple genes frequently mutated in PDAC. These include: *TP53*, *KRAS*, cyclin-dependent kinase inhibitor 2A (*CDKN2A* encodes the p16 (INK4A) and the p14 (ARF) tumor suppressor proteins), and *SMAD4* (encodes the small mothers against decapentaplegic homolog 4 protein, which is a transcription factor). These genes have been determined to have mutations, deletions, amplifications or inactivations for quite some time now. There are other genes which may be mutated or expressed abnormally in PDAC. Some genes that are also more frequently mutated in PDAC include: cyclin-dependent kinase inhibitor 2B (*CDKN2B* encodes p15INK4b tumor suppressor protein) and *ARID1A* (AT-rich interactive domain-containing protein 1A, which is one component of multiple SWItch/Sucrose Non-Fermentable (SWI/SNF) protein complexes that are involved in chromatin remodeling). Mutations at these genes were detected by next-generation sequencing. The effects of the mutations and/or changes in gene expression on the PDAC microenvironment have been recently reviewed [1].

*KRAS* is mutated in >95% of PDACs. The *KRAS* mutations result in the constitutive activation of a critical GTP/GDP GTPase exchange protein which is an important regulator (on/off switch) in multiple signal transduction pathways [2]. *KRAS* mutations can control various aspects of the tumor microenvironment. *KRAS* mutations can influence the presence of various immune cells in the inflammatory PDAC tumor microenvironment. *KRAS* mutations can affect the infiltration of T cells and myeloid-derived suppressive cells (MDSCs) during early stages of pancreatic intraepithelial neoplasia (PanIN) [3]. This can result in changes to pancreatic stellate cells (PSC) and induce mesenchymal-derived cells to form fibroblasts and fibrin remodeling. This results in PDAC remodeling of the microenvironment and progression of the PanIN and the ability of immune cells to infiltrate the PDAC microenvironment [4]. *KRAS* mutations can also increase the expression many genes associated with the immunosuppressive PDAC microenvironment such as the immune check point regulator programmed death-ligand 1 (PD-L1) [5,6]. This can result in the differentiation of CD4^+^CD25^−^ cells into T regulatory (Treg) cells [7] and recruitment in colorectal cancer (CRC), PDAC and other cancers [8]. In addition, *KRAS* mutations induce growth factors such as interleukin-6 (IL-6) and IL-10, transforming growth factor-β (TGF-β) and sonic hedgehog (Shh) [9].

**Figure 1:**
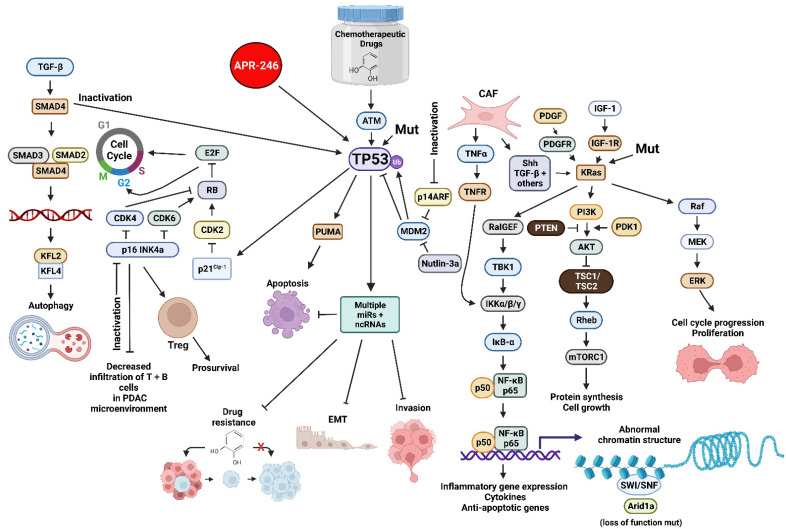
Sites of Interactions of Mutant Genes in PDAC and the Various Pathways which they Effect and also Sites of Interaction of Certain Small Molecule Inhibitors Discussed in this Review. Abbreviations: transforming growth factor-β (TGF-β), small mothers against decapentaplegic homolog 4 protein (SMAD4), small mothers against decapentaplegic homolog 3 protein (SMAD3), small mothers against decapentaplegic homolog 2 protein (SMAD2), Kruppel-like factor 4 (KLF4), Kruppel-like factor 2 (KLF2), cyclin-dependent kinase 4 (CDK4), cyclin-dependent kinase 2 (CDK2), cyclin-dependent kinase inhibitor 2A encoded by *CDKN2A* (p16 INK4a) E2F transcription factor (E2F), cyclin-dependent kinase inhibitor 1A encoded by *CDKN1A* (p21Cip1), small molecule reactivator of mutant TP53 (APR-246), TP53 = tumor suppressor protein 53 KDa, 14 KDa tumor suppressor protein alternate reading frame protein encoded by *CDKN2A* (p14ARF), p53 upregulated modulator of apoptosis (PUMA), mouse double minute 2 E3 ubiquitin ligase (MDM2), small molecular inhibitor of MDM2 (nutlin-3a), micro RNA (miR), non-coding RNAs including LncRNAs and circRNAs (ncRNA), platelet-derived growth factor receptor (PDGFR), cancer-associated fibroblast (CAF), tumor necrosis factor-α (TNFα), tumor necrosis factor receptor (TNFR), sonic hedgehog growth factor (Shh), Ral guanine nucleotide exchange factor (RalGEF), TANK binding kinase 1 (TBK1), inhibitor of NF-Kappa-B kinase-α/β/γ (IKKα/β/κ), inhibitor of NF-κB-α (IκB-α), p50 KDa subunit of nuclear kappa-κB cells (p50), p65 KDa subunit of nuclear kappa-κB cells (p65), small molecule multi kinase inhibitor (Sorafenib), platelet-derived growth factor (PDGF), phosphatase and tensin homolog (PTEN), insulin-like growth factor-I (IGF-1), insulin-like growth factor-1 receptor-1 (IGF-1R), Kirsten Ras oncogene homolog (KRas), phosphatidylinositol 3-kinase (PI3K) 3-phosphoinositide-dependent protein kinase-1 (PDK1), AKT serine/threonine kinase (AKT), (TSC1 (hamartin) and TSC2 (tuberin) tumor suppressor complex (TSC1/TSC2), Ras Homolog, mTORC1 binding protein (Rheb), mechanistic target of rapamycin kinase (mTORC1), SWItch/Sucrose Non-Fermentable complex (SWI/SNF), AT-rich interactive domain-containing protein 1A (Arid1a)—MAP kinase kinase kinase (Raf), mitogen-activated protein kinase kinase 1 (MEK), ERK = mitogen-activated protein kinase (ERK). We compiled the information necessary for all the figures, designed all the figures and their composition was created with BioRender.com.

The *TP53* gene encodes a tumor suppressor protein. The *TP53* gene is one of the most frequently mutated genes in humans. The TP53 protein is a transcription factor. TP53 can also influence the PDAC or CRC microenvironment by influencing the expression of many genes. Inactivation of wild type (WT) TP53 activity has direct effects on cell cycle progression, apoptosis and senescence. Loss of the normal activities of TP53 changes the immune milieu in the PDAC microenvironment and promotes inflammation which is pro-tumorigenic. Mut-TP53 can alter immunosuppressive properties of the PDAC microenvironment which accelerates tumor progression and metastasis [10]. TP53 can increase the immune response in the PDAC microenvironment by augmenting the levels of T cells, which enhances the effects of dendritic cells (DC) [11]. This was determined by treatment with the mouse double minute 2 homolog (MDM2) inhibitor nutlin-3a. WT TP53 suppresses IL-6 expression while it is detected at higher levels in the PDAC microenvironment in cells with mut-TP53. Increased IL-6 expression is associated with metastasis [12]. Mut-TP53 can also induce NF-κB activity, which in turn induces inflammatory cytokine expression, including IL-6 and tumor necrosis factor-α (TNF-α), and promotes metastasis [13]. In colon cancer models, mutant TP53 can induce the expression of vascular endothelial growth factor (VEGF) which promotes angiogenesis and tumorigenesis [14]. An overview of some of these interactions is presented in Figure 1. 

*CDKN2A* is another gene whose expression is frequently altered in PDAC [15]. The *CDKN2A* locus encodes two proteins, p16 (p16/INK4A) and p14ARF. Altered expression (loss of function) of the *CDKN2A* locus can occur by many mechanisms, including promoter methylation and gene deletion. p16/INK4A and p14ARF are normally tumor suppressor proteins and they serve to function to inhibit cell cycle progression. p16/INK4a normally inhibits cell cycle progression by suppressing the activity of cyclin-dependent kinase 4 (CDK4) and CDK6. The p14ARF protein induces cell cycle arrest by inhibiting the degradation of MDM2, which results in destabilizing the TP53 protein. *CDKN2A* mutation (downregulation) has been associated with decreased infiltration of T and B cells in the PDAC microenvironment. This also increased the levels of forkhead box P3 positive (Fox3P) Tregs and was associated with a lower level of survival in PDAC patients [16]. 

SMAD4 is a tumor suppressor protein. The *SMAD4* gene encodes a transcription factor, and it is downstream of TGF-β [17]. Mutation or loss of SMAD4 activity prevents the growth suppressive effects of TGF-β. Loss of SMAD4 activity may result in tumor angiogenesis. Loss of SMAD-4 and WT-TP53 activities were associated with S100A8. The S100A8 protein binds calcium and zinc and regulates various inflammatory and immune reactions. It can induce neutrophil chemotaxis and adhesion [18]. S100A8 is associated with the tumor microenvironment as it increases the secretion of pro-inflammatory cytokines [19]. 

## 2. Interactions of TP53 with the Immune System and Fibroblasts in the PDAC Microenvironment

The immune system is key for the prevention of bacterial infections as well as prevention of abnormal growth and tumor development. Although T and B cells are some of the best-known cells in the immune system, especially the adaptive immune response, the innate immune response is also important in regulating tumor growth. The innate immune system consists of macrophages, monocytes, DC, natural killer (NK) and other cells. The PDAC microenvironment consists of tumor-infiltrating T, B and NK lymphocytes as well as myeloid cells including macrophages, monocytes, DC, MDSC and other cells. There are two types of suppressor/regulatory cells in the PDAC microenvironment: Treg cells and type 2 macrophages (M2, MDSC). M2 macrophages can differentiate into tumor-associated macrophages (TAM) cells. These cells promote tumor inflammation and immunosuppression. The extracellular matrix (ECM) and cytokines/chemokine milieu is altered in the PDAC environment. Inflammatory stress is monitored by TP53, and in the absence of WT TP53 this censoring is diminished.

In studies with TP53 knock-out mice (TP53-null), enhanced levels of IL-1, IL-6 and IL-12 were observed in the macrophages. Increased levels of these proinflammatory cytokines altered macrophage function in the PDAC microenvironment [20]. Inhibition of normal TP53 function led to T cell differentiation into T helper Th17 cells [21]. Loss of WT-TP53 activity altered Treg differentiation and led to inflammation [22,23]. Restoration of WT-TP53 activity suppressed inflammation and autoimmunity [20].

In addition, the PDAC microenvironment has cancer-associated fibroblasts (CAF). CAFs alter the pancreatic cancer microenvironment by the secretion of growth factors such as C-X-C motif chemokine ligand 1 (CXCL1), CXCL12, C-C motif chemokine ligand 8 (CCL8), stromal cell-derived factor-1 (SDF-1), IL-6, IL-11, VEGF and others [24,25,26]. The CAFs can have important effects on immunosuppression, angiogenesis and metastasis in the tumor microenvironment by secreting multiple growth factors. Likewise, primary pancreatic cancers can modulate the tumor microenvironment by secreting various factors in exosomes which favor colonization in metastatic sites [27].

## 3. Interactions between Stroma and TP53 and Their Regulation of miRs, LncRNAs and CircRNAs in the PDAC Microenvironment

PDAC is associated with a dense dysplastic stroma which results in a hypoxic environment and impedes the effectiveness of chemotherapeutic drugs as well as immunotherapeutic approaches. Targeting the dense dysplastic stroma has been attempted but it has not yet proven to be effective and may be counter-productive [28].

CAFs are the main producers of stoma [29]. Hypoxia inducible factor-2α (HIF-2α) is a protein present in the PDAC stroma. Recently, it has been shown that HIF-2α is a potential therapeutic target for PDAC. This may occur by obstruction of the crosstalk between CAFs and macrophages in the PDAC stroma. Belzutifan is an HIF-2α and HIF-1α inhibitor that is approved for the treatment of renal cell carcinoma [30]. The HIF-1α and HIF-2α genes were conditionally knocked out in CAFs that expressed α-smooth muscle actin (α-SMA). In spontaneously arising PDAC tumors, CAFs were isolated, and it was determined that HIF-2α inhibition prevented the crosstalk between the CAFs and macrophages and improved the survival of the mice [31]. PT2399 is an HIF-2α inhibitor that is being examined in pre-clinical studies.

**Figure 2:**
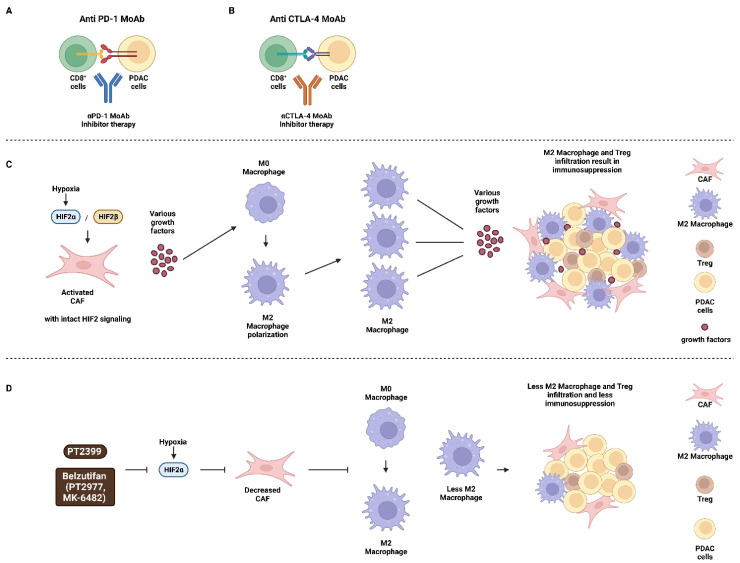
Potential Sites of where Certain Immunotherapeutic Drugs and Inhibitors may Act in the PDAC Microenvironment. Panels (**A**,**B**) immunotherapeutic approaches to target PDAC cells with: (**A**) αPD-1 MoAb and (**B**) αCTLA-4 MoAb. Panels (**C**,**D**) effects of hypoxia on M2 macrophage polarization and the PDAC microenvironment in Panel (**C**), where HIF-2 signaling is intact and Panel (**D**), where HIF-2 signaling is suppressed after treatment with HIF inhibitors PT2399 or Belzutifan. CD8^+^ cells = CD8 positive T cells (CD8^+^ cells), PDAC tumor cells (PDAC cells), monoclonal antibody to programmed cell death protein 1 (αPD-1 MoAb), monoclonal antibody to cytotoxic T lymphocyte antigen-4 (αCTLA-4 MoAb), hypoxia inducible factor-2α (HIF-2α), hypoxia-inducible factor-2β (HIF-2β) small molecule HIF-2α inhibitor (PT2399, PT2977), small molecule HIF-2α inhibitor (Belzutifan, MK-6482).

Macrophages are classified as M1 and M2. M1 macrophages are activated and pro-inflammatory, while M2 macrophages become activated during the resolution phase of inflammation and are immunosuppressive in nature. Treatment of mice with PDAC tumors with PT2399 inhibited macrophage hemotaxis and M2 polarization and improved responses to immunotherapy as well as mouse survival [31]. HIF-2α but not HIF-1α was determined to drive the immunosuppressive environment and result in increased levels of Tregs and TAMs. M2 macrophages were determined to be a major source of M2-polarized TAMs, PD-L1 and cluster of differentiation 86 (CD86), all of which are immunosuppressive molecules. An overview of potential approaches to target the PDAC microenvironment is presented in Figure 2. This figure also presents some of the many cell types and growth factors present in the PDAC microenvironment.

The dense fibrotic stroma present in the PDAC microenvironment impedes drug delivery to the PDAC tumor cells. Various anti-stromal therapies have not yet proven effective in increasing the effectiveness of chemotherapeutic drugs to improve treatment outcomes [28]. The dense fibrotic stroma is complex as it normally impedes PDAC progression, while elimination of the dense fibrotic stroma increases PDAC progression. An important miR involved in various cancers is miR-29. Loss of miR-29 expression results in the activation of PSCs and increased levels of extracellular matrix deposition in the PDAC stroma [32]. In contrast, experimentally induced increased levels of miR-29 expression in activated PSCs cells inhibited stromal deposition, PDAC viability and suppressed growth in in vitro co-culture models. TGF-β1 is growth factor that has tumor promoting as well as fibrotic inducing properties. It is secreted from cancer cells and injured acinar cells. It is important in the activation of PSCs.

**Figure 3:**
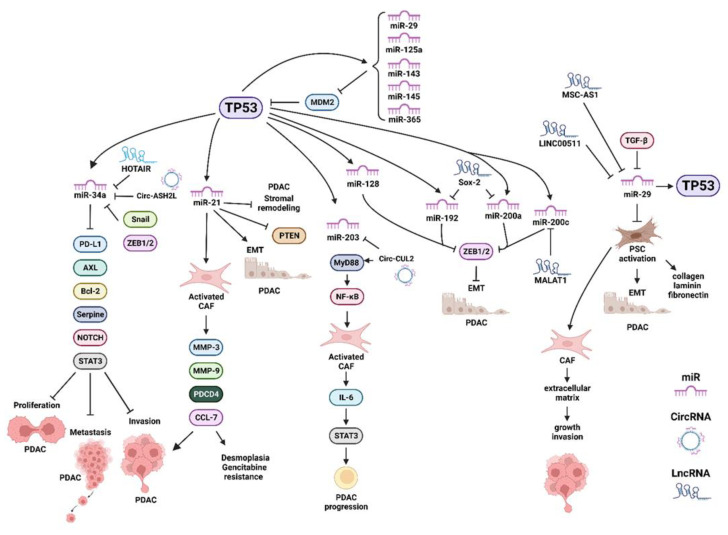
Various miRs, LncRNA and circRNAs Induced by TP53 which have Effects on: Proliferation, EMT, Migration, Invasion, Metastasis, Desmoplasia and Drug Resistance. Programed cell death ligand 1 (PD-L1), B-cell lymphoma-2 gene (Bcl2), AXL = AXL receptor tyrosine kinase, plasminogen activator inhibitor-1 (serpine-1), signal transducer and activator of transcription 3 (STAT3), matrix metallopeptidase 3 (MMP3), matrix metallopeptidase 9 (MMP9), programmed cell death 4 (PDCD4), chemokine ligand 7 (CCL7), MyD88 = myeloid differentiation primary response protein MyD88 (MyD88), IL-6 = interleukin 6 (IL-6), Zinc finger E-box-binding homeobox 1/Zinc finger E-box-binding homeobox 2 (ZEB1/2).

TGF-β1 was determined to be a negative regulator of miR-29 in PSCs. It increased the expression of collagens, laminin and fibronectin, all components of the ECM [33]. Regulatory loops between TP53 and miR-29 exist, as TP53 can activate miR-29 expression and miR-29 can negatively regulate MDM2 expression, resulting in increased stability of TP53 [34]. The effects of multiple miRs, long non-coding RNAs (lncRNAs) and circular RNAs (circRNAs) have been implicated in regulating the PDAC microenvironment and have recently been summarized [35]. A diagram of some of the interactions between TP53 and various miRs, lncRNAs and circRNAs and their effects on the PDAC microenvironment is presented in Figure 3. In this figure, we have indicated where the miRs, LncRNAs and CircRNAs (ncRNAs) affect either the PDAC tumor cells or the CAFs.

The effects of the circCUL2 RNA were examined in cells with an inflammatory CAF phenotype in the PDAC microenvironment [36]. This occurred by a myeloid differentiation primary response protein MyD88 (MyD88)-dependent nuclear factor-κB cell (NF-κB) signaling pathway activation. circCUL2 RNA was detected in CAF phenotype but not in the PDAC tumor cells. circCUL2 RNA was associated with a poor prognosis in PDAC patients. Experimentally increased expression of circCUL2 in normal fibroblasts resulted in cells with an inflammatory (iCAF) phenotype. These cells synthesized IL-6 and were able to stimulate PDAC progression. circCUL2 was determined to act as competing endogenous RNAs (ceRNA). ceRNAs function by binding miRs and inhibiting their function. CircCUL2 inhibited miR-203a-3a and suppressed its ability to inhibit MyD88 and downstream NF-κB and IL-6 and subsequent signal transducer and activator of transcription 3 (STAT3) signaling [36].

PSCs are important in PDAC. They are a subset of CAF [37]. Activated PSC promote PDAC migration via desmoplastic interactions which lead to increased collagen, laminin and other proteins at the extracellular matrix. This contributes to fibrosis. miR-29a has been shown to affect PSCs and alter the regulation of the PDAC microenvironment [32]. miR-29a modulates effectors of IGF-1/TP53 signaling in PSCs. miR-29 may hinder carcinogenesis in PDAC. miR-29a expression is regulated by TP53, and miR-29a in turn regulates TP53 expression by regulating MDM2 [38]. This also increases the resistance to treatment with the chemotherapeutic drug temozolomide. TP53 can induce the expression of miR-29 in certain cancers [34,39].

CAFs are important cells during the desmoplastic reaction in PDAC [40]. Oxidative stress is important in the PDAC tumor microenvironment. However, the origins of CAFs are not well understood. Experimentally, oxidative stress can be induced by either H_2_O_2_ or radiation treatment. Oxidative stress stimulated monocyte-to-myofibroblast transdifferentiation (MMT) of CD14^+^ monocytes. This resulted in increased levels of α-SMA expression. The increased levels of α-SMA expression were dependent on p38^MAPK^ pathway activation. Oxidative stress in the PDAC tumor microenvironment could induce MMT in PDAC. This increased reactive stroma and promoted both immunosuppression and tumor progression. The MMT resulted in the generation of CAFs with reduced phagocytic capacity. Importantly, in this model, the CAFs could promote the proliferation of PDACs. Reducing oxidative stress has been proposed as a therapeutic regimen [41].

**Figure 4:**
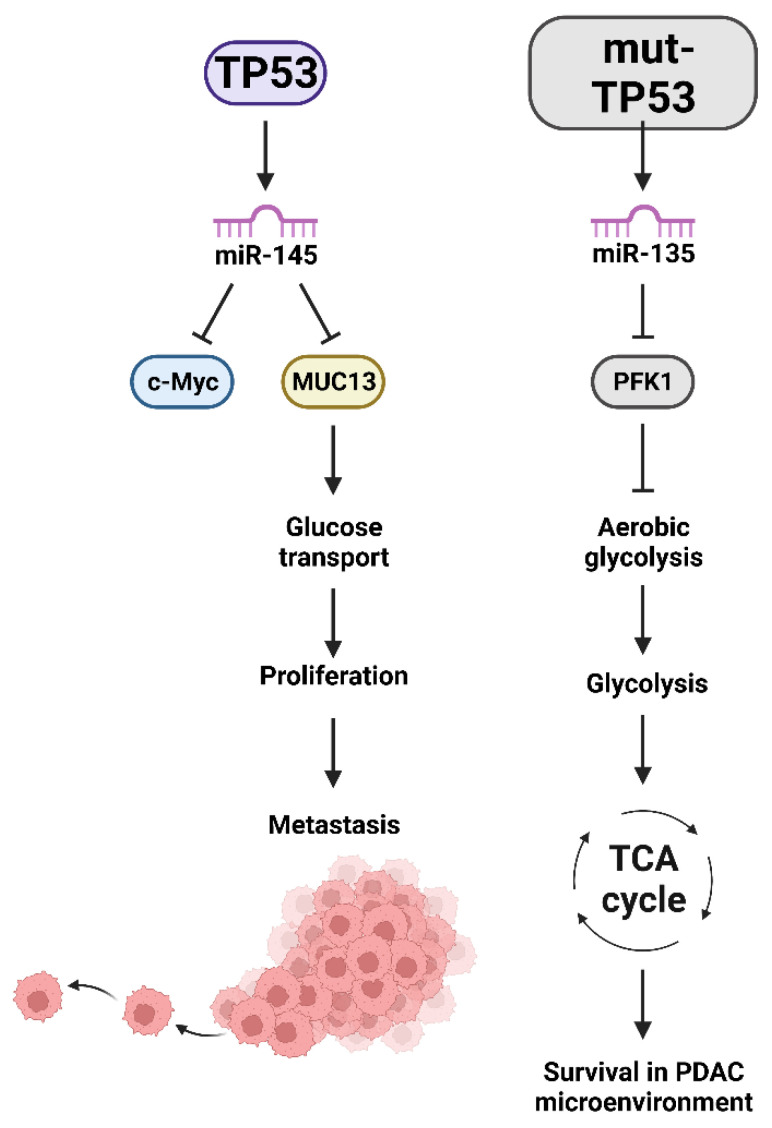
Effects of miRs induced by TP53 which have Effects on Glucose Transport, Aerobic Glycolysis, TCA Cycle, Survival in the Hypoxic PDAC Microenvironment and Metastasis. v-Myc avian myelocytomatosis viral oncogene homolog (c-Myc), mucin 13 (MUC13), PFK1 = phosphofructokinase-1 (PFK1), tricarboxylic acid cycle (TCA).

Tumor-associated stroma (TAS) has been implicated as playing critical roles in the PDAC tumor microenvironment. Exosomes were determined to transfer miR-145 from TAS to PDAC tumors. In this situation, the TAS suppressed tumor growth [42]. miR-145 is regulated by TP53 in various cancers including pancreatic cancer [43]. miR-145 targets multiple mRNAs encoding proteins important in tumor progression. miR-145 functions as a tumor-suppressor in pancreatic cancer and inhibits the expression of the mucin 13 (MUC13) gene [44]. MUC13 increases many events associated with malignant transformation including proliferation, migration and invasion. MUC13 supports multiple signaling pathways in the pancreatic tumor microenvironment. In the absence of WT-TP53, there will be less miR-145 and more MUC13 in the tumor microenvironment. MUC13 also has effects on glucose metabolism in PDAC, which is important for survival of the PDAC cancer in the hostile pancreatic tumor microenvironment [45]. A diagram of these potential interactions is presented in Figure 4.

miR-21 is also important in the PDAC microenvironment [35]. miR-21 is also induced by TP53 and can be secreted by M2 macrophages [35]. In animal studies, loss of miR-21 promoted stromal remodeling and accelerated tumor initiation and progression. This occurred via increased mucinous cystic neoplastic lesions. miR-21 can mediate TGF-β-mediated EMT and stemness [46].

miR-21 has been shown to regulate CAFs activation. The relationship between miR-21 in CAF activation and chemotherapeutic drug resistance was examined in both tumor samples from PDAC patients and tumor transplant studies. PDAC patients that were resistant to gemcitabine had higher amounts of miR-21 and increased levels of CAFs. Increased levels of matrix metalloproteinase-3 (MMP-3), MMP-9, PDGF and CCL-7 were detected in CAFs with high levels of miR-21. These increases promoted invasion in PDAC cell lines. Increased miR-21 expression also regulated programmed cell death 4 (PDCD4) gene expression which resulted in CAF activation. PDCD4 interacts with and inhibits the eukaryotic translation initiation factor 4A1 activity by preventing RNA binding. In the tumor transplant model, it was determined that upregulating miR-21 in CAFs resulted in PDAC desmoplasia and the gemcitabine-drug resistance. In contrast, suppressing miR-21 in CAFs in the tumor transplant studies resulted in inhibition of PDAC desmoplasia and led to sensitization to gemcitabine [47].

miR-21 can also regulate the expression of the PTEN phosphatase which is an important tumor suppressor gene. Decreased expression of PTEN has been observed in the PDAC stromal cells, which may be mediated by miR-21 [48]. TP53 regulates the expression of miR-21 in some cancer types such as hepatocellular carcinoma [49].

miR-194-5p down-regulates the expression of the immunoregulatory checkpoint PD-L1 molecule and alters the PDAC microenvironment. Overexpression of miR-194-5p inhibited the proliferation, migration and invasion of PDAC cells in vitro. In mouse orthotopic PDAC models, miR-194-5p suppressed PDAC proliferation, stimulated the infiltration of CD8^+^ T cells and augmented IFN-γ release by CD8^+^ lymphocytes in the PDAC tumor microenvironment. Thus, miR-194-5p may serve as a therapeutic target in PDAC by altering the tumor microenvironment [50].

miR-128 regulates the cluster of differentiation 47 (CD47) gene which in turn controls the zinc finger E-box-binding homeobox 1 (ZEB1) in PDAC. miR-128 inhibited the proliferation and metastasis of PDAC by increasing the numbers of DCs, CD8^+^ T lymphocytes and NKs and increased anti-tumor immunity [51].

Interactions between TAMs and pancreatic cancer cells are important in PDAC microenvironment. M2 macrophages release exosomes which contain miR-501-3p, which controls transforming growth factor beta receptor 3 (TGFBR3) mediated signaling in PDAC cells, which promotes tumor progression. TGFBR3 is a tumor suppressor gene. miR-501-3p targets TGFBR3. This resulted in activation of the TGF-β signaling pathway, which led PDAC migration, invasion and tumor formation in mouse models [52].

miR-135 is expressed at higher levels in PDAC patient samples than in normal adjacent tissues. Glutamine deprivation results in increased miR-135 expression. This was determined to occur by reactive oxygen species (ROS)-induced activation of mut-TP53. Mut-TP53 was determined to promote miR-135 expression. One of the targets of miR-135 is phosphofructokinase-1 (PFK1). When PFK1 is suppressed, aerobic glycolysis is also inhibited. This results in the cells using glucose to mobilize the tricarboxylic acid (TCA) cycle. Inhibition of miR-135 expression led to the PDAC cells becoming sensitive to glutamine deprivation and suppressed tumor growth. These studies suggest a mechanism by which PDAC cells can exist in the harsh tumor PDAC microenvironment. miR-135 and mut-TP53 confer a loop that allows the PDAC cancer to survive in the nutrient poor PDAC microenvironment and permits the PDAC cells to survive under metabolic stresses [53].

TAMs were determined to release exosomes containing miRs which conferred resistance to the chemotherapeutic drug gemcitabine in the PDAC microenvironment. miR-365 was determined to suppress the activation of gemcitabine by increasing the triphosphonucleotide pool and the enzyme cytidine deaminase. Cytidine deaminase normally inactivates gemcitabine. Treatment with a miR-365 antagonist (antagomiRs) resulted in sensitivity to gemcitabine [54]. In some cases, miR-365 can regulate the expression of TP53 by suppressing the expression of the TP53 regulator MDM2 [55].

miRs are important in the regulation of cytokine expression in the PDAC microenvironment. Various miRs influence the expression of cytokines which inhibit migration in the TAS. TAS is very abundant in the PDAC microenvironment. This is responsible, in large part, to the lethal nature of PDAC [56]. Various miRs were determined to be expressed in PDAC, namely, miR-205, miR-200a, miR-200b, miR-200c, miR-141 and miR-429. This was consistent with an epithelial miR phenotype. In contrast, miR-145, miR-199a and miR-199b were detected in TAS cells consistent with a stromal miR phenotype. When miR-200b, miR200c and miR-205 were over expressed in TAS cells, increased granulocyte macrophage colony stimulating factor (GM-CSF) and interferon gamma-induced protein 10 (IP10) were secreted at higher levels and migration was inhibited [56]. TP53 can regulate the expression of miR-205 [57]. TP53 can also modulate the expression of miR-200, which in turn regulates the expression of transcription factors such as ZEB1 and ZEB2 which promote EMT in certain cell types such as hepatocellular carcinoma [58]. The TP53/miR-200 axis is important in PDAC [59]. The levels of TP53/miR-200 are critical to preventing EMT in PDAC. In the presence of low levels of TP53/miR-200, nuclear factor of activated T cells 1/SRY-box transcription factor 2 (NFATc/Sox2) promote EMT and the “stemness” of pancreatic cancer cells. The NFATc/Sox2 complex promotes the transcription of “stemness”-associated genes such as Snail family transcriptional repressor 1 (*SNAI1*) and ZEB2. miR-200c normally suppresses Sox2 expression as well as certain EMT-associated molecules such as Snail1 and ZEB2. Inactivation of the TP53/miR200 pathways was shown to be essential for the dedifferentiation of PDAC cells.

The expression of miR-141, miR-149 and mi-429 are regulated by TP53 in some cancers such as gastrointestinal cancers including PDAC [60,61]. In summary, many miRs play key roles in the PDAC microenvironment.

## 4. Altered Expression of Growth Factors, Their Receptors and Downstream Signaling Pathways in the PDAC Microenvironment

TP53 is a member of a multigene family which includes TP53, TP63 and TP73. Members of this gene family have different functions and various effects on cellular growth. TP53 GOF mutations can alter the tumor microenvironment in PDAC by allowing the production of growth factors leading to increased proliferation. GOF TP53 mutations can bind and suppress the pro-apoptotic functions of p73 and p63 [62].

**Figure 5:**
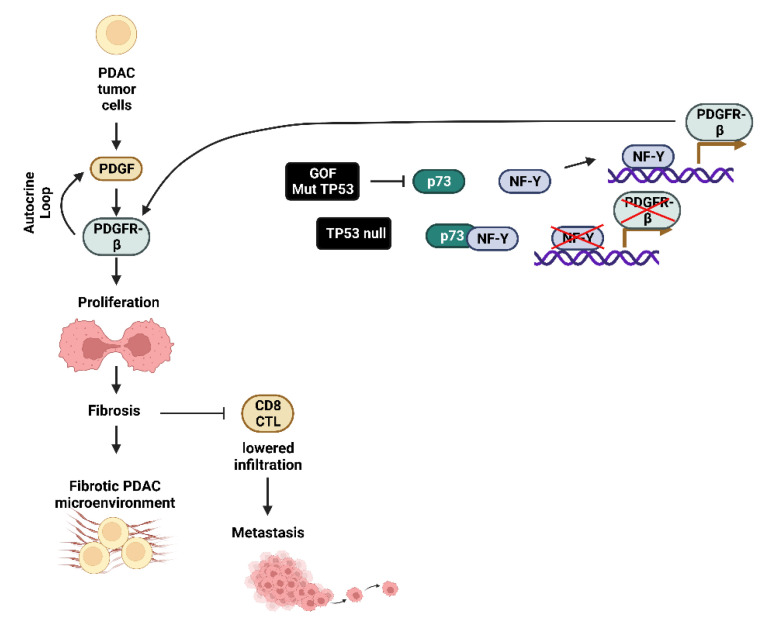
Effects of GOF mut-TP53 on PDGF-Rβ Expression in PDAC Tumor Cells Growth, Fibrosis and Metastasis. CD8^+^ cytotoxic T lymphocyte (CD8^+^ CTL), TP53 family member p73 (p73), nuclear factor-Y transcription factor (NF-Y).

GOF-TP53 mutants can repress the formation of the TP73/nuclear factor Y (NF-Y) transcription factor complex on the platelet-derived growth factor receptor-β (PDGFR-β) promoter region. This allows NF-Y to bind the promoter region of the PDGFR-β gene and stimulate transcription. Autocrine PDGF is produced in the tumor cells; it binds the PDGFR-β and drives PDAC metastasis. In PDAC cells which have deleted *TP53* (TP53-null), TP73 is still able to bind NF-Y, which suppresses PDGFR-β expression. In contrast, in the presence of GOF-TP53, there is increased PDGFR-β expression which results in increased fibrosis and reduced infiltration of cytotoxic CD8^+^ lymphocytes and contributes to metastasis. GOF-TP53 mutations result in a fibrotic tumor microenvironment [63]. This tumor microenvironment suppresses the ability of the immune system to eliminate the PDAC and results in a poor PDAC prognosis [64]. A diagram of these interactions between mut-TP53 and PDGFR-β expression and metastasis is presented in Figure 5.

Autocrine production of PDGF has been observed in various tumor types. Deregulated PDGF/PDGFR-β expression may provide a target for certain PDACs [65,66]. There are already approved small molecule inhibitors such as Imatinib which target the PDGFR-β kinase. In mouse studies, targeting the vascular endothelial growth factor receptor (VEGFR) and PDGFR with dovitinib revealed promising results [67].

Signal transduction pathways are altered in pancreatic cancers and affect various properties of the tumor microenvironment [68]. The abnormal activation of various signal transduction pathways contributes to the pancreatic cancer growth, progression and drug resistance.

**Figure 6:**
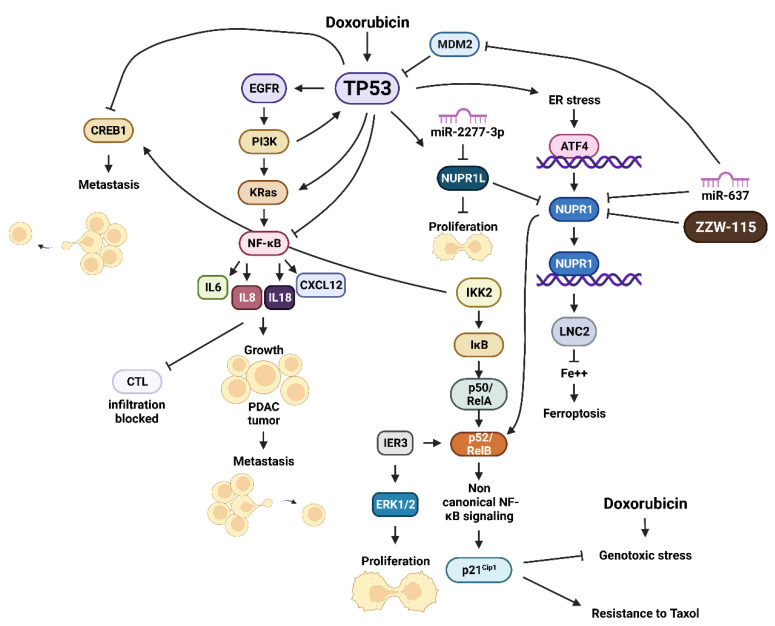
Effects of TP53 Signaling Pathways and Cellular Stress and NUPR1 and NUPR1L Expression. Cyclic AMP response element binding protein1 (CREB1), interleukin 8 (IL8), interleukin 18 (IL18), helix-loop-helix nuclear protein 1 (NUPR1), NUPR1 ligand (NUPR1L), inhibitor of NF-κB kinase-2 (IKK2), inhibitor of NF-κB (IκB), p50 NF-κB/RelA one NF-κB related proteins, v-Rel avian reticuloendotheliosis viral oncogene homolog B (p50/RelA), ATF4 = activating transcription factor 4 (ATF4), lipocalin 2 (LNC2), small molecule inhibitor of NUPR1 (ZZW-115).

Various cytokines, chemokines and growth factors are detected at elevated levels in pancreatic cancer cells. The elevation in expression is often mediated by mutations in *KRAS*, epithelial growth factor receptor (*EGFR*), phosphatidylinositol 3-kinase (PI3K, *PIK3CA*) and *TP53* [69]. These mutations can contribute to elevated levels of the NF-κB signaling which regulates expression of many inflammatory cytokines, including IL-6, IL-8 and IL-18 [70]. The induction of these cytokines leads to an autoregulatory loop which perpetrates NF-κB activation/signaling. The expression of these cytokines contributes to pancreatic cancer progression and metastasis [71]. NF-κB signaling activates the expression of CXCL12 in PSCs. This promotes PDAC tumor growth and inhibits the infiltration of cytotoxic T cells which cannot eliminate the PDAC tumor [72]. A diagram of these interactions is presented in Figure 6.

The TP53 and NF-κB pathways interact and cross-regulate each other. Some of the interactions are altered in PDAC which have mutations at TP53. NF-κB and TP53 may compete for the transcription coactivator p300 and the cyclic AMP response element binding protein1 (CREB1) [73,74]. TP53 can bind the NF-κB promoter region of the p65 subunit. This suppresses p65 expression. In addition, TP53 can suppress the activity of the IκBα kinase (IKKα) [75]. In TP53-null mice, hyperactivation of NF-κB signaling occurs in T cells, macrophages and intestinal epithelium [76].

The JAK/STAT pathway is also activated by cytokines and interferons (IFN). The JAK/STAT pathway has been shown to play critical roles in pancreatic cancer. Elevated expression of the JAK/STAT pathway is associated with a poor prognosis in PDAC [77]. STAT3 hyperactivity is essential for the development of myeloid-suppressor cells. This is important in inflammation and tumorigenesis [78]. TP53 inactivation results in STAT3 hyperactivation in a TP53-null mouse model [79]. Elevated STAT1 expression was observed in the macrophages of TP53-null mice. This resulted in elevated levels of inflammatory cytokines [20]. Loss of WT TP53 activity has different effects on various STAT isoforms. Loss of WT TP53 allows STAT3 to induce the differentiation of Th17 cells. In contrast, WT TP53 can induce STAT5, which can prevent the differentiation of Th17 cells [22].

High JAK2 expression is associated with a poor prognosis in PDAC [80]. IFNα, IFNβ and IFNγ can increase the expression of PD-L1 in pancreatic cancer [81]. Chronic JAK/STAT signaling inhibits the infiltration of cytotoxic T lymphocytes (CTLs) and results in chronic inflammation. Suppression of JAK/STAT signaling by treatment with the JAK inhibitor ruxolitinib increased CTL infiltration in the PDAC microenvironment [82].

The Hippo pathway is very important in the microenvironment of pancreatic cancer. The Hippo pathway is critical in controlling cell growth and organ size. It is a multi-kinase signaling cascade. The Yes-associated protein 1 (YAP) and the transcriptional coactivator with PDZ-binding motif (TAZ) are the main effector molecules in the Hippo pathway. YAP has been shown to promote keys events in KRas-mediated pancreatic cancer tumorigenesis, namely: growth, drug resistance, metabolic reprogramming, differentiation, EMT and metastasis [83,84]. The behavior of PSC can be altered by YAP and TAZ. This alters the recruitment of TAMs and myeloid-derived macrophages in the PDAC microenvironment. This results in the expression of multiple cytokines and chemokines which alter the differentiation and presence of MDSCs in the PDAC microenvironment [85,86]. The YAP and TP53 pathways interact and serve to cross-regulate each other. Aberrant TP53 activity serves to drive YAP-mediated tumorigenesis [87]. YAP can induce the reprogramming of cancer cells into cancer stem cells in some models [88].

The Wnt/β-catenin signaling pathway is important in the PDAC microenvironment as it stimulates EMT and stem-like phenotype, tumor progression and drug resistance [89,90]. TP53 can modulate Wnt signaling via miR-34 [91].

Mut-KRas and mut-TP53 interact to alter the differentiation of PDAC cells, which is mediated in part by Wnt-β-catenin signaling. Mut-KRas activates both the Raf/MEK/ERK and PI3K/Akt signaling pathways, in turn activating CREB1 transcription factor. CREB1 then interacts with mut-TP53 to induce forkhead box O1 (FOXO1) which in turn stabilizes β-catenin signaling and promotes EMT which drives PDAC metastasis. This study also suggests additional targets for PDAC including MEK1, Akt and CREB1 [92]. For some of the molecules, small molecule inhibitors have been developed and evaluated in clinical trials.

In the hypoxic PDAC microenvironment, HIF-2α induces Wnt signaling via β-catenin and SMAD4 and increases tumor progression [93]. Axin2 is a protein which normally inhibits Wnt/β-catenin signaling. The long intergenic non-protein coding RNA 1133 (LINC01133) lncRNA was shown to affect PDAC EMT by suppressing Axin2 expression. LINC01133 was detected in exosomes of advanced PDAC patients and correlated with a poor overall survival [94].

The HOXA transcript at the distal tip (HOTTIP) lncRNA has been shown to stimulate the Wnt/β-catenin pathway in pancreatic cancer stem cells. Increased levels of HOTTIP lncRNAs were detected in PDAC patients and was associated with a poor prognosis and lower levels of survival. The HOTTIP lncRNA interacts by attaching to WD repeat-containing protein 5 (WDR5) which binds to homeobox A9 (HOXA9) locus and induces its expression, which in turn increases the activity of the Wnt/β-catenin signaling. The transcription factor HOXA9 induces the expression of many genes which are associated with stemness and EMT. WDR5 is a component of the histone H3K4 methyltransferase complex and modulates the expression of HOXA9 [95].

## 5. Roles of Hypoxia in the Induction of HIFs, TP53, miRs and LncRNAs in the PDAC Microenvironment

The PDAC microenvironment is hypoxic. In PDAC tumor samples, a positive association was observed between HIF-1α and miR-212 expression. HIF-1α was determined to bind an HIF-1α responsive element in the miR-212 gene by a chromatin immunoprecipitation assay and induced its expression, which in turn increased PDAC growth and metastasis. Knock-down of miR-212 expression suppressed PDAC cell growth in in vitro studies [96]. HIF-1 interacts with the TP53 pathway. Loss of HIF-1α led to increased levels of protein phosphatase 1 regulatory inhibitor subunit 1B (PPRIR1B) and resulted in degradation of TP53, invasion, and metastasis [97].

The pancreatic microenvironment is created by a desmoplastic reaction that results in a dense microenvironment that creates hypoxia and EMT. The microenvironment promotes invasion and metastasis. One LncRNA that is upregulated by hypoxia is the non-coding RNA activated by DNA damage, (NORAD) lncRNA. The expression of NORAD was examined in thirty-three paired cancerous and noncancerous PDAC patient samples by RT-PCR. NORAD was determined to be expressed at high levels in pancreatic cancer tissues and was correlated with shorter overall survival. Increased levels of NORAD promoted migration and invasion, while inhibiting NORAD expression decreased EMT and metastasis both in vitro and in vivo in a mouse orthotopic PDAC model. NORAD may act as a ceRNA to regulate the expression of the small GTP binding protein Ras homolog family member A (RhoA) by competition for hsa-miR-125a-3p which normally inhibits RhoA. This contributes to hypoxia-induced EMT in PDAC [98]. hsa-miR-125a-3p can regulate TP53 activity by inhibiting MDM2 expression, which results in stabilization of TP53 [99].

In experiments with non-small cell lung cancer cells and patient samples, HIF-1α was demonstrated to bind GOF mut-TP53 proteins and regulate the transcription of many genes that were associated with the extracellular matrix. This altered expression of genes promoted tumor progression. GOF mut-TP53 proteins bind HIF-1α, the GOF mut-TP53 affects the transcriptional ability of HIF-1α, and HIF-1α is redirected to other regions of the chromatin. GOF mut-TP53/HIF-1α complex bind the SWI/SNF chromatin remodeling complex. Two prominent ECM components that are affected by this interaction are type VIIa1 collagen and laminin-γ2, which promote tumor progression [100].

GOF mut-TP53 was also shown to affect the chromatin structure in pancreatic cancer cells. This resulted in increased resistance to the chemotherapeutic drug gemcitabine. GOF mut-TP53 induced chromatin remodeling by altering the activity of SWI/SNF chromatin remodeling complex [101]. Assay for transposase-accessible chromatin with high-throughput sequencing (ATAC-seq) resulted in the identification of approximately 500 chromatin sites which were responsible for the changes in gene accessibility. Some of the genes encode proteins known to interact with the TP53 pathway. GOF mut-TP53 induced the expression of macrophage-stimulating protein 1 receptor (MST1r), which is involved in gemcitabine resistance and is sometimes mutated in various cancers. MST1r is the receptor for the macrophage-stimulating protein 1 (MSP1) and is a tyrosine kinase. The promoter region of the MST1r gene contains binding sites for p63, ETS proto-oncogene 2, transcription factor (ETS2), NY-F and sterol regulatory element-binding transcription factor (Srebp) transcription factors. The proteins encoded by these genes interact with TP53. In summary, GOF mut-TP53 regulates gene expression by finely controlling chromatin accessibility.

TP53 and hypoxia have opposing effects on cancer stem cells. HIF-2 will induce the expression of genes which have roles in stem cell function. Octamer-binding transcription factor 4 (Oct-4) is a transcription factor that has been observed to be induced in cancer cells under hypoxic conditions [102,103]. In contrast, TP53 can induce both p21^Cip-1^ and miR-34, and this results in suppression of certain markers associated with stemness such as the transcription factors Oct-4, SRY (sex determining region Y)-box 2 (Sox-2) and Nanog homeobox (Nanog) [104,105].

Hypoxia will also increase the levels of GOF mut-TP53. As stated previously, GOF mut-TP53 has unique properties which have different effects on tumor progression. MCF-7 breast cancer cells are normally WT at TP53. MCF-7 cells transfected with GOF mut-TP53 have increased basal levels of HIF-1α than cells with WT-TP53 [106]. The presence of the GOF mut-TP53 in MCF-7 cells blocks the ability of MDM2 to bind HIF-1α and this results in the stabilization of HIF-1α. GOF mut-TP53 induces the expression of multiple genes and Lnc-RNAs which are important for survival of the cancer cell in the hypoxic microenvironment. TP53 and hypoxia-induced signaling pathways have many interactions which have been reviewed recently [107].

## 6. TP53 as a Regulator of Metabolism in the PDAC Microenvironment

TP53 plays many diverse roles in the regulation of metabolism [108]. Many of biochemical processes are essential for survival of the PDAC cells in the hypoxic tumor microenvironment. Metabolic reprogramming occurs in the PDAC microenvironment which is crucial for PDAC tumorigenesis [109]. In the hypoxic PDAC microenvironment, HIF-1α is stabilized and induces the expression of many genes which are involved in glycolysis such as glucose transporter-1 (GLUT-1) [110]. HIF-1α is overexpressed in PDAC [111]. HIF-1α expression is detected frequently in pancreatic cancers and is associated with their angiogenesis and progression [112].

Hypoxic conditions will induce the expression of two negative regulators of TP53, namely MDM2 and MDM4 in some cell types [113,114]. Hypoxia inhibits TP53 activity by blocking the phosphorylation of the regulatory S15 and S293 on the TP53 protein [115,116].

TP53 can also regulate HIF-1α. TP53 can induce PARKIN gene expression which affects glucose metabolism and inhibits breast cancer progression [117,118]. Parkin is an E3 ubiquitin ligase. Parkin ubiquitinates HIF-1α which results in its proteasomal degradation [119].

HIF-1β is a constitutively-expressed molecule which forms a heterodimer with HIF-1α. The expression of HIF-1β is not regulated by hypoxia, but it can be regulated by TP53. TP53 can induce the expression of miR-107 which inhibits HIF-1β expression. This will prevent the formation of HIF-1α/HIF-1β heterodimers and suppresses angiogenesis of colorectal cancer [120]. TP53 and hypoxia have opposite effects on angiogenesis. TP53 normally acts to inhibit angiogenesis while HIF-1α promotes angiogenesis. HIF-1α can induce VEGF and PDGF, two critical growth factors involved in angiogenesis in various cancer types [121,122,123].

A well characterized phenomenon in cancer is the Warburg effect. The Warburg effect results in increased glucose uptake, enhanced glycolysis and lactate production. This occurs by increased expression of glucose transporters, and glycolytic enzyme including enolase 1, hexokinase 1/2 phosphoglycerate mutase and pyruvate kinase [124,125,126]. Mut-TP53 can increase the expression of GLUT-1 by inducing its translocation to the plasma membrane [127]. HIF-1α also increases GLUT-1 levels [128] as well the expression of glycolysis-related genes [129]. PDAC cells can survive in the hostile microenvironment by undergoing metabolic reprogramming. This results in altering their energy metabolism.

Interestingly, topotecan is a chemotherapeutic drug that is used to treat certain types of cancer patients such as ovarian cancer patients. Topotecan treatment decreased HIF-1α levels in ovarian cancer patients which have GOF mut-TP53 [130]. While many cancer patients have mut-TP53, there are mutant TP53 reactivators that will restore some of the activities of WT-TP53. Some mutant TP53 reactivators have been examined in clinical trials and are employed in the treatment of certain cancer types such as adult acute myeloid leukemia (AML) and myelodysplastic syndromes (MDS) with mut-TP53. It could be relevant to examine the effectiveness of combining topotecan and mutant TP53 reactivators on the treatment of PDAC cells and patients. It would be interesting to determine if topotecan and mutant TP53 reactivators prevent the development of the hostile tumor microenvironment in PDAC models.

TP53 regulates the expression of many genes involved in glycolysis. TP53 can often regulate these genes via miR-34a. Some of these genes include hexokinase 1 (HK1), hexokinase 2 (HK2), glucose-6-phosphate isomerase (GPI), and lactate dehydrogenase a (LDHA) [131,132]. As TP53 is frequently mutated in PDAC and low levels of miR-34a are associated with a poor prognosis [133]. As mentioned previously, miR-143/miR-145 can regulate the expression of TP53 by targeting MDM2 which results in the stabilization of TP53 (WT and GOF mut-TP53). miR-143/miR-145 can also target many genes important in glycolysis including HK2 [134]. Oncogenic KRas, which is present in virtually all PDACs, represses miR-143/miR-155 expression and this could contribute to the PDAC tumor microenvironment [135]. Thus, the expression of these genes in glycolysis could be altered by mutations present in *TP53* and *KRAS*. This could contribute to the metabolic reprogramming in the PDAC tumor microenvironment.

GOF mut-TP53 proteins have been associated with increased expression of the mevalonate (MVA) pathway in some cancers such as breast cancer [136]. The mevalonate pathway is required for the synthesis of cholesterol and nonsterol isoprenoids. WT-TP53 has been shown to suppress the MVA pathway in mouse models of liver cancer [137]. In cells with WT-T53, the activation of the master transcription activator of the MVA pathway, sterol regulatory element-binding protein-2 (SREBP-2) is blocked by TP53 inducing the ATP-binding cassette transporter (ABCA1) cholesterol transporter gene. In this scenario, ABCA1 is acting as a tumor suppressor protein. In mouse cells lacking WT-TP53, this tumor suppression is lost and there is more activated SREBP-2 and transcription of genes such as 3-hydroxy-3-methylglutaryl-CoA reductase (HMGCR), lanosterol synthase, (LSS), squalene monooxygenase (SQLE) and mevalonate metabolites such as mevalonate 5-phosphate (MVAP), geranylgeranyl pyrophosphate (GGPP), and cholesterol are elevated. This increases the activity of the MVA pathway, and tumors develop. Statins serve to block the effects of activation of the MVA pathway. Both WT-TP53 and GOF mut-TP53 interact with SREBP-2, they have different effects as WT-TP53 represses the mevalonate pathway, while GOF mut-TP53 stimulates the MVA pathway.

The MVA pathway is altered in many cancer types including pancreatic cancer. Increased MVA pathway activity is associated with increased protein prenylation which is linked with a malignant cell phenotype. This results in increased invasion and cell survival. When the MVA pathway is at elevated levels, stabilization of the GOF mut-TP53 protein occurs which promotes protein prenylation and enhances cancer progression [138]. The MVA pathway is important in colon cancer cells which lack WT-TP53 by increasing ubiquinone synthesis essential for maintaining mitochondrial electron transport. This is required in metabolically comprised environment such as colon spheroid cultures and colon tumor organoids for respiration and pyrimidine synthesis [139].

TP53 can regulate ferroptosis which is important in the PDAC microenvironment. TP53 can induce or suppress ferroptosis depending on the cell context [140]. The small molecule inhibitor MMRi62 was initially characterized as a molecule which disrupted the activity of MDM2 and MDM4 and could induce apoptosis in a TP53-independent fashion in PDAC both in vitro and in vivo [141]. MMRi62 induced cell death in PDAC by increased autophagy, reactive oxygen species and lysosomal degradation of nuclear receptor coactivator 4 (NCOA4) and ferritin heavy chain (FTH1) which is characteristic of ferroptosis. MMRi62-induced proteasomal degradation of mutant TP53 occurred in PDAC cells with mut-KRas and either double or single mutated TP53. MMRi62 prevented metastasis of PDAC in orthotopic mouse models by the induction of ferroptosis which inhibited cell migration and invasion [141]. The small molecule mutant TP53 reactivator APR-246 can also induce ferroptosis in acute myeloid leukemia cells which have mutant TP53 [142].

Autophagy is a macrometabolic process. Autophagy plays important roles in pancreatic cancer and the PDAC microenvironment [143]. The TP53 gene status was shown to have a critical role in autophagy in PDAC [144]. In a humanized genetically-modified mouse model of PDAC, the TP53 gene status was shown to play a pivotal role in tumor development [144]. Mice that contained an activated KRAS gene mutation develop precancerous PanIN lesions, some of which turn into PDAC. Mice which also lacked the autophagy-related gene 5 (ATG5) or ATG7 genes accumulated PanIN but did not progress to high-grade PanIN and PDAC. In mice lacking WT-TP53 but containing mutant KRAS, loss of autophagy did not block tumor growth but accelerated tumor onset by increasing glucose uptake and anabolic pathways which accelerated tumor growth. Treatment of mice containing mutant KRAS and lacking WT-TP53 with the autophagy inhibitor hydroxychloroquine accelerated tumor growth. Autophagy-induced metabolic cross talk in the PDAC microenvironment between non-cancer cells and cancer cells [144]. Various miRs and LncRNAs are involved in the regulation of autophagy; these include miR-506 [145], miR-372 [146] and LncRNA PVT1 [147].

The mut-KRas protein can be released during autophagy-dependent ferroptosis. This induced uptake of mut-KRas proteins by TAMs and results in the conversion of the TAMs into M2-macrophages. This phenotype was associated with a poor prognosis in PDAC patients [148].

## 7. Interactions between TP53 and the Stress-Inducible NUPR1 Oncoprotein

Various types of cellular stress including oxidative, endoplasmic reticulum (ER) and metabolic stress will activate the helix-loop-helix nuclear protein 1 (NUPR1) and TP53 [149,150,151,152]. The NUPR1 and TP53 proteins play critical roles in cancer [153].

The NUPR1 protein can interact with TP53 in immortalized breast epithelial cells [154]. One of the targets of the interaction between NUPR1 and TP53 was the transcriptional upregulation of p21^Cip-1^. The chemotherapeutic drug doxorubicin upregulated the expression of TP53, p21^Cip-1^ and NUPR1 in MCF-10A immortalized breast epithelial cells. In MCF-10A cells, NUPR1 was determined to bind the p21^Cip-1^ promoter in cells transfected with NUPR1; in contrast, when the cells were transfected with a dominant negative TP53 construct, the binding to the p21^Cip-1^ protein was suppressed. NUPR1 bound and coimmunoprecipitated with the TP53 protein. These interactions resulted in the prevention of genotoxic stress induced by doxorubicin in immortalized breast epithelial cells. MCF-10A cells expressing NUPR1 were also more resistant to the chemotherapeutic drug taxol than MCF-10A not expressing NUPR1.

The expression of NUPR1 was determined to be upregulated in acute pancreatitis and, at even higher levels in pancreatic adenocarcinoma [155], NUPR1 can also interact with mut-KRas signaling in pancreatic cancer and other cells. Genetic studies indicated that in the absence of NUPR1 in a mouse model, mut-KRAS (G12D) did not result in the development of PanIns, which is a precursor of the high lethal PDAC [155,156]. NUPR1 protected the pancreatic cancer cells from apoptosis by a RelB-mediated non-canonical NF-κβ pathway which interacted with immediate early response 3 (IRE3) protein. Subsequently, it was determined that NUPR1 interacted with mut-KRas in the induction of senescence-associated gene networks [157]. NUPR1 was determined to regulate DNA methyltransferase 1 (DNMT1) expression and genome DNA methylation and mut-KRas-induced senescence. The DNA methylation inhibitor 5-aza-2’-deoxycytydine could reverse mut-KRas-induced PanIN progression by inducing senescence [158]. An overview of the interactions between TP53 and NUPR1 is presented in Figure 6.

IRE3 was determined to be responsible for an activation of ERK1/ERK2 by inhibiting protein phosphatase 2A (PP2A) dephosphorylation, which resulted in proliferation and PDAC development [159]. The PDAC tumors which developed in the NUPR1 knock-out mice expressed higher levels of various markers associated with stemness such as aldehyde dehydrogenase 1 (ALDH1), Sox2 and Oct-4 than in mice containing WT-NUPR1 [160].

An important event which happens in chronic pancreatitis and pancreatic cancer is pancreatic fibrosis. Fibrosis is due to the deposition of the ECM and collagen fibers. This occurs due to the necrosis which develops as an attempt to repair the damaged pancreas tissue. Fibrosis is believed to be a critically important driver in promoting the transition of chronic pancreatitis into pancreatic cancer. NUPR1 may be a critical transcription factor which is induced in response to the cellular stress and could be a therapeutic target in pancreatic cancer progression [161].

Recently, NUPR1 has been determined to play critical roles in ferroptosis in PDCA [162]. NUPR1 was determined to have effects on lipocalin-2 (LCN2) expression, and this blocked ferroptosis by decreasing iron accumulation and oxidative damage. Depletion of LNC2 in LNC2-conditional knock-out mice had similar effects as NUPR1 depletion. Interestingly, restoration of LNC2 conferred resistance to ferroptosis induced by erastin. Thus, NUPR1 is an important regulator of iron metabolism and the induction of ferroptosis which is important in the PDAC microenvironment.

ZZW-115 is a NUPR1 inhibitor which induces necroptosis [163,164]. Suppression of NUPR1 or LNC2 by either sh-RNA or ZZW-115 increased the sensitivity to the ferroptosis inducer erastin. Suppression of NUPR1 or LNC2 worsened pancreatitis in mouse models.

ZZW-115 has been examined for its effects on PDAC. ZZW-115 treatment of mice bearing pancreatic tumor xenografts resulted in a dose-dependent decrease in tumor size. NUPR1 was determined to bind importins, a group of proteins involved in the transport of proteins containing a nuclear localization signal (NLS) into the nucleus. ZZW-115 was determined to inhibit the cytoplasmic to nuclear translocation of NUPR1. ZZW-115 competes with the binding of importins to NUPR1 and thus NUPR1 remains in the cytoplasm. ZZW-115 bound the NLS region of NUPR1 [164].

Further studies demonstrated that ZZW-115 could sensitize PDAC cells to genotoxic agents including chemotherapeutic drugs 5-fluorouracil, oxaliplatin and gemcitabine as well as γ-radiation both in vitro and in vivo. ZZW-115 treatment was determined to suppress the SUMOylation of several key proteins involved in the DNA damage response (DDR). Further studies indicated that recombinant NUPR1 protein could enhance the SUMOylation in a cell free system. Thus, NUPR1 may serve to stimulate SUMOylation of key proteins involved in the DDR, and ZZW-115 inhibits this SUMOylation [165].

There is also a *NUPR1*-related gene which is called *NUPR1L*. NUPR1L is a member of the high-mobility group (HMG) family of proteins which can translocate into the nucleus and bind DNA. A function of NUPR1L is to bind and downregulate the activity of NUPR1 promoter, suppress NUPR1 expression and inhibit cell proliferation. The expression of NUPR1L is regulated by TP53 as there are two TP53-responsive elements in the *NUPR1L* gene. Chemotherapeutic drugs such as oxaliplatin can induce TP53 as well as NUPR1L expression [166].

There is a miR which targets NUPR1L. miR-2277-3p targets NUPR1L, and when NUPR1L is suppressed, proliferation, migration, and invasion of SW620 CRC cells occurred [167].

The role of NUPR1 has also been examined in hepatocellular carcinoma (HCC) [168,169]. The levels of NUPR1 are very low in healthy liver tissue. Higher levels of NUPR1 are observed in later stages of HCC progression. The effects of ZZW-115 on two HCC cell lines (HepG2 and Hep3B) were examined by both in vitro and xenograft models. ZZW-115 was determined to induce cell death by both apoptotic and necroptotic mechanism. This treatment resulted in lower ATP production and mitochondrial metabolism failure [170].

The effects of knock down (KD) of NUPR1 were examined in HCC cell lines [168,170,171]. Normally, sorafenib treatment of HCC cells results in activation of autophagic flux. KD of NUPR1 was associated with increased ubiquitin-binding protein p62 (sequestosome-1) expression and decreased autophagic flux and increased sensitivity to sorafenib. In NUPR1 KD cells, increased levels of the TP53 family member p73 were detected as well as downstream targets including PUMA, NOXA and p21^Cip-1^. When both NUPR1 and p73 were silenced, increased resistance to sorafenib was observed in comparison to when only one of the two was inhibited. NSC59984 is a p73 activator. When p73 was activated by NSC59984 and combined with sorafenib, a synergistic inhibition of tumor growth was observed in HCC xenograft models. These results suggest a potential novel approach to treat HCC patients [171].

## 8. Possibility of Treatment of PDAC with Small Molecule Signal Transduction Inhibitors

Although preclinic studies with various signal transduction inhibitors have yielded interesting results, clinical trials have not yet indicated enhanced efficacy in PDAC patients. Some of the kinase inhibitors examined affect the activities of more than one kinase, which may make the therapy difficult to interpret. In addition, it may be necessary to also target more than one mutant protein in the PDAC.

*KRAS* is mutated in >90% of PDAC patients, so multiple attempts to develop KRas inhibitors were undertaken. However, there were problems with the specificity of the initial Ras inhibitors. Today, better mutant KRas inhibitors have been isolated that are clinically used [172,173,174]. LUMAKRAS™ (Sotorasib, AMG510) has been approved by the FDA and used to treat patients with non-small cell lung cancer (NSCLC) who have the G12C *KRAS* mutation. Adagrasib (MRTX849) is also approved by the FDA to treat patients with NSCLC who have the G12C *KRAS* mutation.

Sorafenib is a multi-kinase inhibitor and one of its targets is PDGFR. It is approved by the FDA to treat many diseases such as hepatocellular carcinoma [175]. Clearly, elucidation of the effects of combining the novel KRas inhibitors and inhibitors which target PDGFR such as sorafenib could yield promising results in PDAC therapy, especially with regards to the tumor microenvironment.

Using a conditional oncogenic KRas mouse model, it was demonstrated that extinction of oncogenic KRas signaling resulted in KRas-independent escaper populations. In the escaper population, a Smarchb-Myc network resulted in mesenchymal reprogramming and independence from Raf/MEK/ERK (MAPK) signaling. The *SMARCB1* gene encodes the SNF5 subunit of the SWI/SNF chromatin remodeler complex that can inhibit Myc. Depletion of Smarcb1 activated the Myc network which resulted in an anabolic shift which resulted in increased protein metabolism and activation of ER stress and unfolded protein response [176]. These results indicate that combining inhibitors which suppress ER stress with chemotherapy might be an effective approach to treat PDAC.

Basal-like mesenchymal PDAC represents a class of PDAC which is advanced and difficult to treat. Recently, it was shown treating this class of PDAC with a MEK (trametinib) inhibitor and the multi-kinase (nintedanib) inhibitor which inhibits mutant KRas signaling allowed the infiltration of cytotoxic and effector T cells, and the cells became sensitive to the immune checkpoint inhibitor PD-L1. The combined inhibitor treatment resulted in cell cycle arrest and cell death, remodeled the immunosuppressive cancer cell secretome, and sensitized the PDAC to immune checkpoint therapy [177].

Autophagy is upregulated in mut-KRas PDAC. PDAC growth and metastasis is dependent on blockade of autophagy. Autophagy can be inhibited by the anti-malarial drug chloroquine. Recently, it was shown that combining chloroquine with a MEK inhibitor was very effective in suppressing PDAC growth [178]. This synergistic interaction was demonstrated both by in vitro studies and patient-derived xenograft (PDX)/PDAC models.

Further studies by this same group indicated that suppression of both insulin-like growth factor-1 receptor (IGF-1R) and ERK signaling synergized with autophagy inhibitors to inhibit the growth of PDAC [179]. By performing a CRISPR-Cas9 knock out screen, IGF-1R was determined to be upregulated in response to treatment with autophagy inhibitors. Knock out of IGF-1R augmented autophagic flux and sensitivity to autophagy inhibitors. Downstream of IGF-1R is the KRas/Raf/MEK/ERK/p90Rsk cascade. However, most PDAC cells have activating mutations in *KRAS*, so suppression of IGF-1R by itself is not optimal to inhibit growth with autophagy inhibitors. Thus, combining chloroquine with small molecule IGF-1R (BMS-754807) and ERK (SCH772984) increased the growth inhibition of PDAC cell lines and organoids.

Various approaches to increasing TP53 activity have been examined. Nutlin-3a is an MDM2 inhibitor. It has fewer effects on MDM4. MDM2 and MDM4 both inhibit TP53. In some cells, they are expressed at different levels [180]. They can work independently or in concert to inhibit TP53. MDM2 will add a solo ubiquitin moiety (monoubiquitination) to the TP53 protein. MDM2 can heterodimerize with MDM4. When MDM2 is heterodimerized with MDM4, polyubiquitination of TP53 occurs [181]. NSC207895 (XI-006) is an MDM4 inhibitor. MDM2/MDM4 are ubiquitin ligases. The peptide ATSP-7041 is a dual MDM2/MDM4 inhibitor. MDM2/MDM4 normally targets TP53 for proteasomal degradation. Inhibition of MDM2/MDM4 results in stabilization of TP53. The effects of nutlin-3a on PDAC have been examined. Various MDM2/MDM4 inhibitors and their presence in clinical trials have been summarized [182]. Certain MDM-2 inhibitors (e.g., PXN822) will synergize with topoisomerase inhibitors (etoposide) to induce cell death in PDAC cells in a TP53-independent fashion [183].

Introduction of a WT-TP53 gene into PDAC cells which have either GOF mut-TP53 or were TP53-null will increase their sensitivity to many chemotherapeutic drugs, signal transduction inhibitors and natural products [184,185]. A suboptimal concentration of nutlin-3a or BBR can increase the sensitivity to many of the chemotherapeutic drugs, targeted therapeutics and nutraceuticals in many cancer types [186,187].

Certain commonly prescribed drugs can affect TP53 activity. The anti-type 2 diabetes drug metformin will induce AMP-activated protein kinase (AMPK), which has effects on TP53 activity in some cells [188]. Metformin can influence the expression of the TP53/miR-34a axis via activation of NAD-dependent deacetylase sirtuin-1 (SIRT1) [189]. In some experiment systems, metformin can interact with TP53 and have protective effects on lung endothelial cells [190]. Metformin can affect the sensitivity of ovarian cancer cells which are either mut-TP53 or TP53-null to the poly (ADP-ribose) polymerase (PARP) inhibitor, olaparib [191]. Decreased levels of AMPK activation (P-AMPK) were detected in PDAC samples as compared to normal tissues. The decreased levels of P-AMPK were associated with a poor prognosis and dense stromal reaction. The dense stromal reaction is a characteristic of PDAC; it consists of increased growth of dense fibrous tissue surrounding the tumor. Metformin treatment was determined to decrease the levels of fibrogenic cytokines produced by PDACs. The cytokines were determined to suppress paracrine-mediated PSC activation. In a PDAC xenograft model, metformin prevented tumor growth. Moreover, metformin treatment increased the effectiveness of gemcitabine. This resulted in inhibition of the desmoplastic reaction [192].

Metformin can also influence the sensitivity of PDACs cells to gemcitabine and alter tumorigenesis by altering the PSCs in the tumor microenvironment. This was determined in a genetically-modified mouse model, LSL-KrasG12D/+; Trp53fl/+; Pdx1-Cre (KPC). Combined metformin and gemcitabine treatment reduced Shh expression and altered the recruitment of tumor supportive PSCs, which reduced angiogenesis [193]. Metformin can also affect the invasive abilities of PDAC cells by blocking TGF-β signaling in the tumor microenvironment [194].

Metformin treatment of three different PDAC cell lines did not inhibit proliferation significantly with concentrations up to 1000 nM [195]. However, addition of lower doses of metformin did lower the concentrations of many chemotherapeutic drugs, signal transduction inhibitors and natural products, indicating that metformin lowered the inhibitory concentration 50 (IC_50_) of these compounds (concentrations necessary to inhibit cellular proliferation by 50%) [195].

In addition, a low dose of metformin could interact with chemically-modified berberines (NAX compounds) and lower the concentrations of berberine (BBR) and certain NAX compounds required to reach the IC_50,_ indicating that metformin could increase drug sensitivity in terms of proliferation [196].

Recently, it was demonstrated that the peroxisome proliferator activated receptor-delta (PPARδ) is upregulated in PanIN. Activation of PPARδ by either a high fat diet or a highly-selective synthetic PPARδ ligand accelerated PDAC development in KRASG12D mutant mice [197]. Activation of PPARδ resulted in the secretion of C-C motif chemokine ligand 2 (CCL2) from KRASG12 cells and led to the recruitment of myeloid-derived suppressor cells into the pancreatic tumor microenvironment via CCL2 and C-C motif chemokine receptor 2 (CCR2) [197]. This drove PanIN progression into PDAC. These results indicated that PPARδ maybe be a novel target for PDAC treatment

Various natural products can activate TP53. BBR is a nutraceutical that has been used in traditional medicine for hundreds of years. It is an isoquinoline quaternary alkaloid (a 5,6-dihydrodibenzo[a,g]quinolizinium) derivative [198]. The effects of BBRs and NAX compounds on PDAC have been recently described [199,200]. Furthermore, the effects of nutlin-3 can be enhanced in PDAC cells by low doses of certain NAX compounds [201].

APR-246 is a mut-TP53 reactivator which has been examined in clinical trials. It has been approved by the FDA for the treatment of myelodysplastic syndrome (MDS) patients who have mut-TP53 in combination with azacitidine, a nucleoside analog which has previously been used to treat MDS patients [202].

Low doses of APR-246 reduced the IC_50_ concentrations of chemotherapeutic drugs, signal transduction inhibitors, BBR, or certain NAX compounds [203,204]. The ability of APR-246 to lower the IC_50_s of chemotherapeutic drugs, signal transduction inhibitors, BBR and modified NAX compounds was dependent on the presence of WT-TP53 or GOF mut-TP53 as APR-246 did not lower the IC_50_s of chemotherapeutic drugs, signal transduction inhibitors, berberine or certain NAX compounds in PDAC cells which were TP53-null.

## 9. Summary

In this review, the importance of TP53, KRas and miRs on various important processes and cells of the PDAC microenvironment has been summarized. Clearly, PDAC development is dependent on more than a single mutation and also more than just PDAC tumor cells in the PDAC microenvironment. Other cell types including immune effector, regulatory T cells, macrophage cells, fibroblastic cells and tumor-associated stroma are involved in regulating PDAC progression and the sensitivity to therapeutic approaches. Moreover, multiple signaling pathways and miRs are aberrantly regulated in PDAC. In addition, fundamental biochemical processes such as metabolism are re-wired in the PDAC tumor environment which makes the PDAC tumor able to survive in the hypoxic environment. The more we learn about the PDAC microenvironment, the more we discover how complicated it is and reasons why it has been so difficult to effectively treat this deadly cancer.

## Data Availability

Not applicable.
